# Plasma Corticotropin-Releasing Factor Receptors and B7-2^+^ Extracellular Vesicles in Blood Correlate with Irritable Bowel Syndrome Disease Severity

**DOI:** 10.3390/cells8020101

**Published:** 2019-01-30

**Authors:** Shin-ichiro Hagiwara, Burcu Hasdemir, Melvin B. Heyman, Lin Chang, Aditi Bhargava

**Affiliations:** 1 The Osher Center for Integrative Medicine, University of California, San Francisco, CA 94143, USA; hagi114@wch.opho.jp (S.-I.H.); burcu@dreadnought.org (B.H.); 2 Department of OBGYN, University of California, San Francisco, CA 94143-0556, USA; 3 Department of Pediatrics, University of California, San Francisco, CA 94158, USA; mel.heyman@ucsf.edu; 4 G. Oppenheimer Center for Neurobiology of Stress and Resilience Vatche and Tamar Manoukian Division of Digestive Diseases David Geffen School of Medicine, University of California, Los Angeles, CA 90095-7378, USA; LinChang@mednet.ucla.edu

**Keywords:** cell-to-cell, B-cells, dendritic cells, EV cargo

## Abstract

Extracellular vesicles (EVs) are composed of bilayer membranes that are released by different cell types and are present in bodily fluids, such as blood, urine, and bile. EVs are thought to play a key role in intracellular communication. Based on their size and density, EVs are classified into small, medium, or large EVs. Cargo composition in EVs reflects physiological changes in health and disease. Patients with irritable bowel syndrome (IBS) exhibit visceral hypersensitivity and mood disorders. Stressful episodes often precede disease symptoms in IBS patients. Stress-induced symptoms include, but are not limited to, abdominal pain and mood swings. Perceived stress responses are mediated by two known G protein-coupled receptors (GPCRs), corticotropin-releasing factor receptor 1 and 2 (CRFRs). CRFRs belong to the Class B secretin receptor family of GPCRs. Here, we show that CRFRs were present in human and murine plasma, and in EVs purified from mouse serum. CRFRs were present in plasma from IBS patients and healthy controls. EVs secreted from immune cells influence both adaptive and innate immune responses via exchange of EVs between different immune cell types. B7-2 (CD86), a plasma membrane antigen-presenting protein, is present on EVs secreted from dendritic, B-, and mast cells, whereas CD9 is present on EVs secreted from dendritic and intestinal epithelial cells. We found that plasma CRFR levels positively correlated with B7-2+ EVs (R = 0.8597, *p* < 0.0001), but no association was seen with CD9+ EVs. Plasma CRFRs expression negatively correlated with IBS severity scores. Our data suggests that plasma EVs from immune cells carry CRFRs as cargos and influence cell-cell communication in health and disease.

## 1. Introduction

Extracellular vesicles (EVs) are composed of bilayer membranes that are released by different cell types. Based on their biogenesis, size, or densities, EVs are classified into different categories [1]. EVs formed from the membranes of intracellular compartments via the endocytic pathways are referred to as exosomes. Exosomes range from 50–100 nm in diameter with buoyant densities of 1.11–1.19 g/mL [1]. Microvesicles that originate from the outward blebbing of the plasma membrane are often referred to as ectosomes or microparticles and range from 100–1000 nm in diameter. The recent guidelines posted by the International Society for Extracellular Vesicles recommends referring to EVs based on their size and densities as small (sEVs), medium, and large (m/lEVs) EVs [2]. EVs are involved in the regulation of a myriad of biological activities, from pro-coagulant to immunomodulation, including tissue repair and remodeling [3]. EVs are being explored as potential biomarkers, drug candidates, drug carriers, or therapeutic targets.

In cultured inner medullary collecting duct (IMCD3) kidney cells, actin-dependent machinery regulates ectosome release from the tip of the cilia. These ectosomes contain G protein-coupled receptors (GPCRs) that fail to migrate from cilia back into the cell [4]. In HEK293 cells, EVs have been shown to contain GPCR heteromers that can be transported between cells [5]. Immune cells can exchange EVs, thereby influencing both innate and adaptive immune responses [6]. T-cell activation can be induced by dendritic cell-secreted EVs that express co-stimulatory molecules, such as CD80 (B7-1) and CD86 (B7-2), or directly by EVs derived from ICAM-1-presenting mature dendritic cells [7,8]. Chemokines and cytokines secreted into the blood by T-cells and monocytes are largely present in EVs [9] and greater than 50% of cytokines secreted from the placental villous tissue are in soluble form, where those secreted by amnion were largely EV-associated [9]. Other bodily fluids and tissues secrete cytokines as a combination of EV-associated or soluble forms. Whether a given cytokine will be present in free-form or EV-associated depends on the source of origin, thereby contributing to heterogeneity. 

Irritable bowel syndrome (IBS), a gut-brain disorder, is classified into three subgroups diarrhea predominant (IBS-D), constipation predominant (IBS-C), and mixed bowel habit (IBS-M) [10]. IBS patients report heightened visceral sensitivity and often suffer from mood disorders. The hypothalamic-adrenal-pituitary (HPA) axis, the system at the core of the stress response, is involved in the modulation of mood behavior. The classic “flight or fight” stress response involves the actions of the corticotropin-releasing factors (CRF) system. The CRF family is comprised of four known agonists, CRF, and three urocortins (UCN1-3), and two known G protein-coupled receptors (GPCRs), CRF_1_ and CRF_2_. CRF_1_ activation initiates HPA responses and anxiety-like behavior, whereas CRF_2_ activation brings systems back to homeostasis, and mediates anxiolytic behavior [11,12]. We have shown that CRF_2_ plays a vital role in mediating metabolic [13] and immune responses [14,15,16,17,18,19]. CRF_2_ receptor dysfunction is known to exacerbate edema, necrosis, and delayed healing [14,20]. Psychological stressors downregulate expression of CRF_2_ receptors [21] and CRF_2_ receptor null mice (Crhr2^−/−^) are more anxious than wild-type littermates [22,23]. CRFRs modulate immune function and permeability in the gut [15,24,25]. The CRF system also modulates visceral hypersensitivity [26] and gut motility in animal models of stress [27]. It is also implicated in the modulation of gut motility and mood behavior in IBS patients [28]. We previously documented increased levels of CRFRs in duodenal biopsies of patients with Crohn’s disease [15], but the status of CRFRs in IBS patients is unknown.

CRFRs belong to the Class B secretin receptor family of GPCRs. Various cell-types and tissues synthesize and express CRFR [15,16,18,24,29,30]. CRFR mediate both autocrine and paracrine responses [31], and thus, it is reasonable to speculate that they might be secreted in to the circulation from various cells and tissues. However, the presence of CRFRs in plasma or EVs has not been shown before. Depending on their origin, CRFRs might be secreted in free form or be EV-associated.

Human EVs are known to contain cytokines, microRNAs, specialized lipid, and proteins [32]. The EV lipid and cargo composition can substantially differ from that of the parental cells, from which EVs originate. EVs are known to change dynamically in number and cargo content in response to physiologic and pathophysiologic milieu [33]. EVs are present in many tissues and bodily fluids. Increased concentration of EVs in bile associates significantly with biliary stenoses and has been proposed as potential markers [34]. EVs present in blood have been studied intensely, but EVs in blood of patients with gut-brain disorders, including IBS patients, have not been reported. Here, we report the presence of stress receptors, CRFRs, in plasma of IBS-D patients and age-and sex-matched healthy controls. Plasma CRFRs in conjunction with B7-2^+^ EVs that might originate from immune cells are potential novel markers for IBS severity and important for cell-to-cell communication contributing to IBS pathogenesis. 

## 2. Materials and Methods

### 2.1. Human Subjects 

Study approval was obtained from the University of California, San Francisco (UCSF, IRB# 12-09375) and University of California, Los Angeles (UCLA, IRB# 12-001731) Institutional Review Boards (IRBs). Subject recruitment at UCSF: UCSF IRB approved the retrospective analysis of these samples and data and a waiver of consent was granted. All control subjects (n = 8; 4/sex) underwent endoscopies for gastrointestinal symptoms (such as abdominal pain), but had no clinical or laboratory findings to support a diagnosis of IBS or inflammatory bowel disease (IBD), as shown in Supplementary Table S1. Patients with any previously diagnosed autoimmune or inflammatory conditions (such as arthritis, thyroiditis, celiac, diabetes, and others) were excluded. Pathology blocks were obtained and paraffin-embedded sections were stained for CRF receptor immunoreactivity, as described below. Subject recruitment at UCLA: Male and female Rome III-positive IBS patients [10,35] and healthy controls (HC) between the ages of 18 and 55 participated in the study. Current IBS symptom severity was measured using the validated IBS Severity Scoring System (IBS-SS, Table 1) [36], and usual severity was measured as none, mild, moderate, and severe [37]. We selected a homogenous patient population (IBS-D) that was age and sex matched to healthy controls; IBS-D patients did not have active psychiatric disease and were not on any prescription medications. Human blood samples were collected in heparin vacutainer at UCLA between August 2013 and April 2017 after informed consent was obtained. Cell debris and platelets were removed from plasma samples by centrifuging it 1500× *g* for 15 min as per the manufacturer’s specifications, and aliquots were stored at −80 °C until further analysis. Our IBS-D patients and healthy controls were well matched and blood samples collected and processed in an identical manner. Thus, sample variability should be the minimum. 

### 2.2. Animals

All animal procedures were approved by the Institutional Animal Care and Use Committee (IACUC approval # AN177899-01) at UCSF and were conducted in accordance with the National Institutes of Health Guide for the Care and Use of Laboratory Animals. Crhr2^−/−^ (C57BL/6 background) mice were a generous gift from Dr. Mary Stenzel-Poore, Oregon Health Sciences University. The mice were housed in a room that was temperature (22–25 °C) and light (12-h: 12-h light/dark cycle starting at 7:00 AM) controlled. Mice had *ad libitum* access to standard Purina chow and water and were handled daily to avoid handling act as a stressor. Crhr2^+/−^ (heterozygous) mice were bred to obtain wild-type (WT) and Crhr2^−/−^ littermates. Serum and/or plasma from WT and Crhr2^−/−^ littermates were used for detection of EVs and serum was used to purify EVs.

### 2.3. Chemicals and Antibodies

All chemicals used were molecular biology grade and from known vendors. The following primary and secondary antibodies were used in this study: CRF-RI/II (Santa Cruz Biotechnology, Dallas, TX, USA; sc-1757; goat polyclonal; 1:1000; recognizes both CRF_1_ and CRF_2_ receptors), CRF-RI (Santa Cruz Biotechnology; sc-12381; goat polyclonal; 1:1000) B7-2 (Santa Cruz Biotechnology; sc-19617; mouse monoclonal; 1:1000), and CD9 (Santa Cruz Biotechnology; sc-13118; mouse monoclonal; 1:1000). For Western blot analyses, secondary antibodies used were goat anti-mouse conjugated to Alexa Fluor 680 (Invitrogen Inc., Carlsbad, CA, USA) and goat anti-rabbit conjugated to IRDye 800 (Rockland Immunochemicals, Pottstown, PA, USA) (both used at 1:20,000).

### 2.4. Extracellular Vesicles Isolation

Extracellular vesicles (EVs) were isolated using ExoQuick exosome precipitation kit (System Biosciences, Palo Alto, CA, USA) according to the manufacturer’s specifications [38,39]. Briefly, 100 μL of serum was mixed thoroughly with 25.2 μL of ExoQuick exosome precipitation solution and incubated for 30 min at 4 °C. The mixture was centrifuged at 1500× *g* for 30 min, supernatant collected, and re-centrifuged at 1500× *g* for 5 min. EV pellets were resuspended in PBS, and total protein concentration was measured using BCA assay (Bio-Rad, Hercules, CA, USA). Six (6) µg of purified EVs per lane were used for immunoblotting. Several factors can influence the use of EVs as biomarkers [40]. The viscosity of bodily fluids can be highly variable, which may affect EVs purity and yield [1,40]. Factors such as age, sex, ethnicity, body mass index, disease, use of medications, general lifestyle, and dietary habits can all influence EV yield and purity. 

### 2.5. Western Blot Analysis

Mice and human plasma were diluted to 1:100 with PBS. Diluted plasma (10 µL) and exosomes (5 μg) were heated at 80 °C for 10 min in sample loading buffer (0.5M Tris-Hcl [pH 6.8], 99% Glycerol, SDS, Bromophenol Blue), resolved on a 10% SDS-PAGE, and transferred to polyvinylidene difluoride membranes (PVDF, Immobilon-FL; Millipore, Billerica, MA, USA). The membranes were blocked for 1 h at room temperature (Odyssey Blocking Buffer; Li-COR Biosciences, Lincoln, NE, USA) and incubated with simultaneously with two primary antibodies at a time for 2 h at room temperature (CRF-RI/II and B7-2 or CRF-RI/II and CD9). Membranes were washed for 30 min × 3 (1x PBS, 0.1% Tween20) and incubated with appropriate secondary antibodies for 1 h at room temperature. Blots were visualized and bands quantified using the Odyssey Infrared Imaging System (Li-COR Biosciences). However, for densitometric analysis of each band/antibody, the colors were separated using a software program that is part of Odyssey’s Li-COR. For quantification of individual bands from immunoblots, the densitometric analysis was done using the Li-COR software by drawing boxes of the same size/area around the bands.

### 2.6. Immunohistochemistry

Immunohistochemistry was performed, as described previously [15]. Five-micron sections from paraffin-embedded biopsy samples were obtained from the duodenum and the colon [15]. Sections were de-paraffinized in xylene and rehydrated in ethanol series and antigen retrieval was performed by the heat method. Sections were incubated with primary antibodies (anti-CRF-RI/II) overnight at 4 °C, washed, and incubated with fluorescent secondary antibodies (conjugated to FITC) for 1 h at room temperature. Confocal images were captured on a Zeiss LSM Meta 510 confocal microscope using Plan-Apochrom at 40× or 63× oil immersion objectives (NA 1.4). Omission of primary antibody served as negative control. 

### 2.7. Statistical Analysis

Statistical analysis was performed using the Prism v7.0 software (GraphPad Software Inc., La Jolla, CA, USA). Two-way ANOVA was used to analyze main effects and interactions between groups. When main effects were significant, Sidak’s multiple comparisons test was used to compare four groups. Tukey’s multiple comparisons test was used to compare CRFR expressions between three usual scores (Severity; Mild, Moderate and Severe). Differences with *p* < 0.05 were considered statistically significant. Data are shown as mean ± standard error of mean (SEM).

## 3. Results

### 3.1. CRF Receptors Are Present in Human Plasma

**Figure 1:**
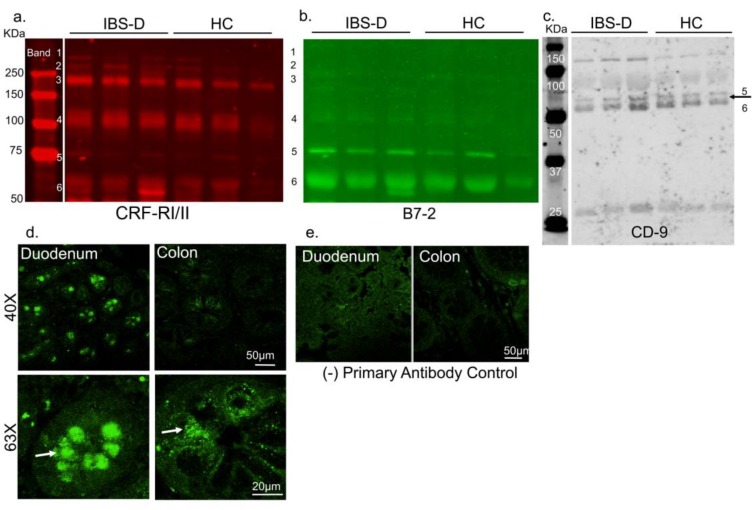
CRF receptors in human plasma, EVs, and human gut. (**a**) Representative immunoblots showing presence of CRF receptors 1 and 2, B7-2, and CD9 bands in plasma of healthy human subjects and IBS-D patients. Band 5 and 6 of CRFR overlapped with bands in (**b**) B7-2, and (**c**) CD9 immunoblots. IBS-D: irritable bowel syndrome patients with diarrhea; HC: healthy controls. (**d**) Representative confocal micrographs showing localization of CRFR granules in human duodenal and colonic biopsies. Intestinal crypts with CRFR immunoreactivity in goblet cells (arrows) at 40× and 63× magnification are shown. Scale: 50 µm at 40× and 20 µm at 63×. (**e**) Representative images of negative controls. Primary antibody for CRF-RI/II was excluded, but all other steps were performed in parallel with other sections shown in 1d.

The presence of GPCRs in circulation is a novel and powerful concept. We first determined that CRFRs were present in human plasma from healthy individuals and patients with IBS-D. Immunoblot of diluted (1:100) serum samples revealed the presence of multiple CRFR bands; six distinct band sizes for were observed (Figure 1a). CRFRs are known to be post-translationally modified, as well as forming heteromers, and thus appear at multiple sizes [41]. Presence of B7-2 bands were also noted, including at its expected size of ~75KDa (Figure 1a). Interestingly, anti-B7-2 (Figure 1b) and anti-CD9 (Figure 1c) also showed multiple bands, including bands that overlapped with CRFR bands 5 and 6, suggesting that EVs that express anti-B7-2 and CD-9 might carry cargos of varying sizes and densities, including CRFRs.

#### 3.1.1. CRF Receptors Are Present in Human Gut

We have recently shown presence of CRF receptors on mast cells [18], enteric neurons, pancreatic acinar cells [16], and fibroblasts [29]. We have also shown increased mast cell infiltration in the gut in a rat model of functional dyspepsia, a gut-brain disorder [19]. While presence of CRF receptors has been shown in colonic tissues of patients with Crohn’s disease [15], it is not known if CRF receptors are also expressed in other gut regions in healthy human subjects. Furthermore, while CRF_2_ receptor transcripts are reported, presence of CRF_1_ receptor in different gut regions is controversial. We reasoned that goblet cells are secretory and release contents into circulation, thus, might be another potential source for secreted CRFRs. Confocal microscopy confirmed that CRFRs were present as punctate bodies in the goblet cells in human duodenal and colonic biopsies (Figure 1d,e). These data suggest that CRFR protein is present in the gut and the protein can both be made locally or secreted from these gut cells into circulation either in free-form or as EV cargo. Since IBS-D patient show visceral hypersensitivity, it is possible that CRFRs released from the goblet cells of IBS patients might have altered function.

#### 3.1.2. CRF Receptors Are Present in Mouse Plasma and Purified EVs

**Figure 2:**
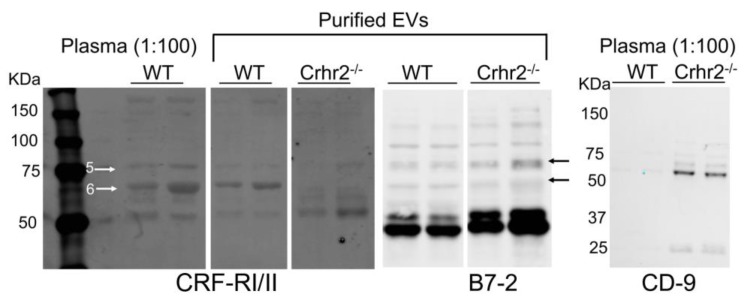
CRF receptors in murine plasma and purified EVs. Representative immunoblots showing CRFR expression and CD9+ EVs in plasma (1:100 diluted) of wild-type (WT) and Crhr2 null mice. Purified EVs from serum show similar CRFR banding pattern in WT mice, whereas Crhr2 null lacked bands at positions 5 and 6 (arrows) that were also seen in human plasma. B7-2 was detected at its expected size of ~75KDa and other bands that overlapped with CRFR, suggesting cargo-related changes in weights and sizes of EVs.

Next, we reasoned that if CRF_2_ receptors are secreted in circulation, then plasma from CRF_2_ receptor knockout mice (Crhr2^−/−^) should not carry any detectable CRF_2_ receptors, but would still express CRF_1_ receptors, whereas plasma from wild-type (WT) mice should express both CRFRs. Immunoblotting revealed the presence of multiple CRFR bands in plasma from wild-type, but not Crhr2^−/−^ mice (Figure 2). Importantly, actin, a housekeeping protein, was present in plasma samples from both WT and Crhr2^−/−^ mice (Supplementary Figure S1). Most GPCRs appear as smears on immunoblots or exist as multiple bands due to several post-translational modifications. We have shown that CRFRs can exist as heteromers manifested as multiple bands, whereas individually transfected CRF_1_ receptors show only subsets of bands [41]. We next performed immunoblot on plasma from WT and Crhr2^−/−^ mice using a CRF_1_ receptor antibody. In agreement with previous published studies, this CRF_1_-specific antibody detected three bands in mouse plasma (Supplementary Figure S2). 

To ascertain that CRFRs are secreted into blood as EV cargoes, EVs from mice serum were purified, and EV markers anti-B7-2 and anti-CD9 [42] were used to confirm identity. B7-2, a plasma membrane antigen-presenting protein is present on EVs secreted from immune cells including dendritic, B-, and mast cells, whereas CD9 is present on EVs secreted from dendritic and intestinal epithelial cells [42]. B7-2 and CD9 were chosen as markers of EVs, because CRFRs are present in immune and intestinal cells, respectively. CRFRs in purified EVs co-localized with B7-2, but not CD9 bands. CRFR expression in EVs from Crhr2^−/−^ mice was considerably reduced (Figure 2).

#### 3.1.3. CRF Receptors are Present in Plasma of Patients with Irritable Bowel Syndrome (IBS) and Associate with EVs and Disease Severity Scores

**Figure 3:**
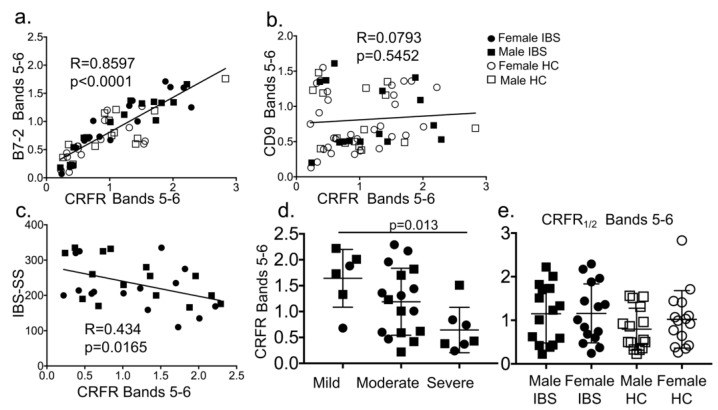
CRF receptor levels show a strong positive association with B7-2+ EVs and a negative relationship with IBS scores. (**a**) Linear regression analysis showed a strong positive correlation (R = 0.859, *p* < 0.0001) between B7-2 and CRFR expression in plasma from IBS-D patients and HC (n = 15/sex/group). (**b**) No correlation was seen between CD9 and CRFR expression in plasma from IBS-D patients and HC (n = 15/sex/group). (**c**) A negative correlation between IBS severity score (IBS-SS) and CRFR expression (R = 0434, *p* = 0.0165) was seen (n = 30 IBS patients). (**d**) CRFR expression decreased in accordance with disease severity of IBS (mild: 1.64 ± 0.56 vs. severe: 0.64 ± 0.44, *p* = 0.013). Statistical analysis: Tukey’s multiple comparisons test. (**e**) CRFR expression was quantified from immunoblot and Two-way ANOVA showed no significant main effect of disease (IBS vs. HC), or sex (n = 15/sex/group).

In plasma from IBS-D patients and healthy controls (HC) (n = 60, demographic and IBS severity described in Table 1), six distinct CRFR bands were detected, as seen for mouse plasma. Two of the six CRFR bands overlapped with B7-2+ and CD9+ EVs (Figure 1a–c). CRFR bands 5–6 that overlapped with B7-2 showed a strong positive association (R = 0.8597, *p* < 0.0001, Figure 3a). Interestingly, CRFR bands 5–6 showed no association with CD9 (R = 0.0793, *p* = 0.5452, Figure 3b). These data suggest that specific immune cells secrete CRFR in EVs. CRFR bands 5–6 in human plasma that overlapped with B7-2 showed a negative correlation with IBS Severity Score (R = 0.434, *p* = 0.0165, Figure 3c) and disease severity (mild, moderate vs. severe, *p* = 0.013, Figure 3d). Criteria for IBS Severity Score [36] and disease severity [37] has been described in these earlier publications. No statistically significant main effect or interaction between IBS and sex or IBS and CRFR bands 5–6 was seen (Figure 3e).

#### 3.1.4. Plasma CRF Receptor Levels Associate with IBS Disease, but Not Sex

**Figure 4:**
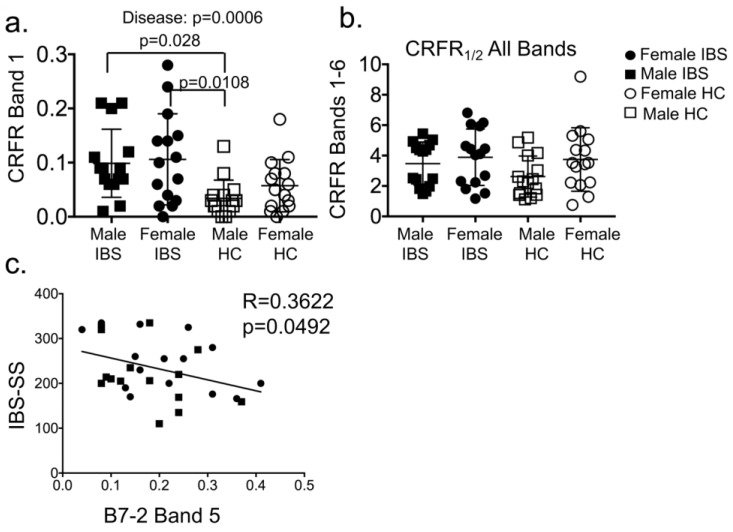
(**a**) Quantification of CRF-RI/II band 1 from immunoblots using plasma of healthy controls (HC) and IBS patients (n = 15/group). Two-way ANOVA showed significant main effect of IBS (*p* = 0.0006), but not sex, and no interaction between IBS (disease) and sex was noted. CRFR Band 1 levels increased in male and female IBS patients compared with male HC (Sidak’s multiple comparisons test; n = 15/group). Male IBS vs. Male HC, *p* = 0.028 and Female IBS vs. Male HC, *p* = 0.0108. (**b**) Quantification of CRFR Bands 1–6 (total bands) from immunoblots from plasma of healthy controls (HC) and IBS patients (n = 15/group). Two-way ANOVA showed no significant main effect of IBS or sex. No significant difference in total CRFR levels between IBS and HC were noted. (**c**) Linear regression revealed a significant inverse association between IBS-SS and B7-2 band 5 (*p* = 0.0492) with an increase in disease severity resulting in decreased B7-2 levels.

Women are twice as likely to suffer from IBS than men. IBS patients show visceral hypersensitivity, and CRF system is involved in the regulation of visceral pain. We tested whether CRF receptor expression was different between healthy individuals and those with IBS and whether sex was a biological variable. Two-way ANOVA showed significant main effect of disease (IBS) on expression of CRFR band 1 (F (1,56) = 13.3; *p* = 0.0006), but surprisingly, no interaction between IBS and sex was noted (Figure 4a). Expression levels of CRFR band 1 were significantly higher in IBS vs. HC. Levels in male and female IBS-D patients were higher compared with male HC (Figure 4a). Total CRFR levels (bands 1–6) did not differ between IBS patients and HC (Figure 4b), whereas bands 1, 5, and 6 did. B7-2 levels were found to inversely correlate with IBS severity score (Figure 4c).

## 4. Discussion

We report several novel observations. First, stress receptors and CRFR are secreted and are present in murine and human plasma. CRF receptors are G protein-coupled receptors (GPCRs) and our report is the first to show presence of GPCRs in circulation. Second, while B7-2+ and CD-9+ EVs might be secreted from several sources, the important finding is that B7-2+ EVs levels and CRFR levels show a strong association in human plasma samples, whereas no association was found with CD-9+ EV. Third, B7-2+, CD9+, and CRFR band compositions in plasma is similar to that in purified EVs, thereby allowing for retrospective detection of EVs in already collected plasma samples. 

CRFRs overlap with EVs potentially secreted from immune cells such as mast cells, B cells, and dendritic cells. Mast cell dysfunction is thought to be involved in modulating visceral hypersensitivity in IBS patients. CRFR levels were significantly higher in plasma of IBS patients compared with HC, and were significantly associated with IBS severity. Presence of GPCRs in circulation opens up the possibility for more nuanced modulation of signaling via these receptors. While in vitro studies that use transfected GPCRs have shown the presence of GPCRs in exosomes [5,43], presence of GPCRs in EVs from in vivo samples had not been demonstrated. These published studies showed that, in cultured cells, GPCRs can be transferred by exosomes from a source cell to a target cell and maintain functionality. Our in vivo data suggested that circulating EVs are potential reservoirs for CRFRs and can be transported from multiple sources to several target tissues for highly nuanced signaling and immune responses.

Several bands for CRF receptors were present in both human and mouse plasma in this study. Crhr2 null mice express normal levels of CRF_1_, and the anti-CRF-RI/II antibodies that cross-reacts with both CRF_1_ and CRF_2_ receptors detected multiple bands, as before [41]. CRFRs from different organs are differentially post-translationally modified [44] resulting in several bands. Various bands are also attributed to CRFRs heteromerization [41]. CRF_2_ receptor, but not CRF_1_ traffics to the plasma membrane from endoplasmic reticulum in actin-dependent manner [41]. In light of the findings that other GPCRs, such as somatostatin are sequestered into ectosomes from the cilia plasma membrane [4], it is plausible that CRF_2_ expressed from the goblet cells in the gut may also be secreted into ectosomes in actin-dependent manner. The observation that total CRFR levels (bands 1–6) did not differ between IBS patients and HC (Figure 4B), whereas bands 1, 5, and 6 did, suggesting to us that these CRFR bands harbor different modifications and may be secreted from different sources/cell types. CRFR may be post-translationally modified differently in IBS patients compared with HC. In agreement with this hypothesis, differential glycosylation of CRFR is reported in health and disease [44]. CRFRs from specific sources contribute to variability in those band sizes and/or are secreted in the plasma in either free-form or in EVs. In support of this notion, a recent study reported that cytokines secreted from the placental villous are largely soluble, whereas those secreted from T-cells, monocytes, and amnion are EV-associated [9]. Thus, blood and bodily fluids harbor a heterogeneous mix of free and EV-associated cargo.

In light of our findings of a strong association of CRFRs with B7-2+, but not CD9+-containing EVs in IBS patients and healthy controls, it would be interesting to further interrogate the composition of B7-2+ EVs by co-immunoprecipitation followed by mass spectrometry. Specifically, mast cells, B cells, and dendritic cells from IBS patients and healthy subjects can be sorted and purified B7-2+ EVs can be characterized further. A study showed that glioma-derived exosomes exerted their effects on monocyte maturation to suppress T-cell immune responses [45]. The cargo composition of these glioma-derived exosome was not delineated, nonetheless, irrespective of the method used to isolate these exosomes, the functional outcome was that the exosomes exerted their effect by acting on monocytes rather than direct interaction with T cells [45]. Thus, the precise nature of EV-associated cargo that alters function, has not been delineated. The paradoxical observation that higher CRFR levels are associated with lower IBS severity scores suggests that CRFRs from immune cells are protective and as the disease progresses, immune cells are unable to make sufficient CRFRs. Indeed, B7-2 levels were inversely correlated with IBS severity score (Figure 4c). It is also possible that the composition of CRFR in EVs between HC and IBS may differ. The lack of association between CRFRs and CD9+ intestinal EVs is intriguing, but this does not rule out the possibility that CRFRs may still be subjected to differential subcellular localization [15] or post-translational modification in the gut of IBS patients vs. HC. 

## 5. Conclusions

Plasma CRFR and B7-2+ EVs in conjunction can serve as potential biomarkers for disease severity in IBS-D patients. Further studies are needed to determine the association with other IBS subtypes and to characterize CRFR-containing and B7-2+ EV composition.

## Figures and Tables

**Table 1:** Characteristics of IBS and healthy controls (HC).

	**Male IBS**	**Female IBS**	**Male HC**	**Female HC**
	**(n = 15)**	**(n = 15)**	**(n = 15)**	**(n = 15)**
Age (years)	27.2 ± 1.613	27.07 ± 3.113	27.53 ± 1.756	27.47 ± 1.082
IBS-SS	221.2 ± 17.47	246.3 ± 15.92		
Usual Severity (n)				
Mild	4	2		
Moderate	8	9		
Severe	3	4		

## Supplementary Materials

The following are available online at http://www.mdpi.com/2073-4409/8/2/101/s1.
