# Supplementary material for: Plasma Corticotropin-Releasing Factor Receptors and B7-2+ Extracellular Vesicles in Blood Correlate with Irritable Bowel Syndrome Disease Severity
Source: Cells. 2019 Jan 30;8(2):101. doi: 10.3390/cells8020101 (PMC6406316; doi:10.3390/cells8020101)
Supplement: Supplementary file 1 [file cells-08-00101-s001.pdf]

Hagiwara et. al

Supplementary Materials:

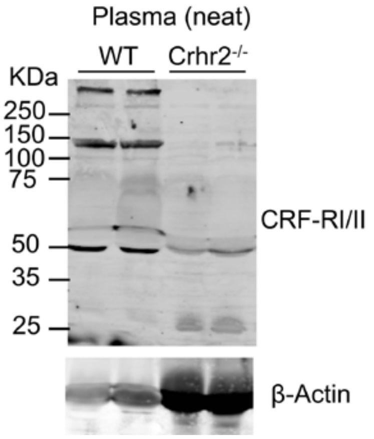

Supplementary Figure 1. CRF receptors in murine plasma from WT mice, but not Crhr2$^{-/-}$ mice. Representative immunoblots showing CRFR expression in plasma (neat) of wild-type (WT), whereas no band specific for CRFR was detected in plasma from Crhr2 null mice. β-actin, a housekeeping gene was present in robust quantities in plasma of WT and Crhr2 null mice.

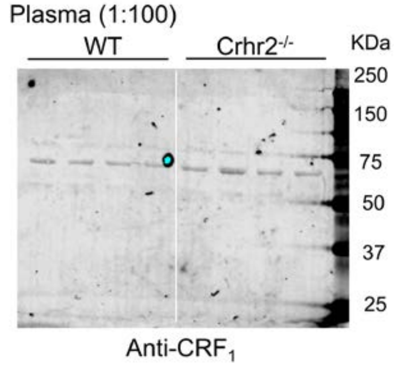

Supplementary Figure 2. Presence of CRF_1 receptors in mouse plasma. Representative immunoblots showing presence of CRF_1 receptor expression in plasma from WT and Crhr2$^{-/-}$ mice. Unlike the CRF-RI/II antibody, this CRF-RI antibody detected only 3 bands, and as expected, expression of CRF_1 receptors in Crhr2$^{-/-}$ mice was not affected.
